# Sequencing individual genomes with recurrent genomic disorder deletions: an approach to characterize genes for autosomal recessive rare disease traits

**DOI:** 10.1186/s13073-022-01113-y

**Published:** 2022-09-30

**Authors:** Bo Yuan, Katharina V. Schulze, Nurit Assia Batzir, Jefferson Sinson, Hongzheng Dai, Wenmiao Zhu, Francia Bocanegra, Chin-To Fong, Jimmy Holder, Joanne Nguyen, Christian P. Schaaf, Yaping Yang, Weimin Bi, Christine Eng, Chad Shaw, James R. Lupski, Pengfei Liu

**Affiliations:** 1 Department of Molecular and Human Genetics, Baylor College of Medicine, One Baylor Plaza, Houston, TX 77030 USA; 2 Human Genome Sequencing Center, Baylor College of Medicine, Houston, TX USA; 3 Baylor Genetics, Houston, TX USA; 4 Instituto de Referencia Andino, Bogotá, Colombia; 5 Department of Pediatrics, University of Rochester Medical Center, Rochester, NY USA; 6 Department of Pediatrics, Baylor College of Medicine, Houston, TX USA; 7 Department of Pediatrics, University of Texas Health Science Center, Houston, TX USA; 8 Institute of Human Genetics, Heidelberg University, Heidelberg, Germany; 9 Department of Statistics, Rice University, Houston, TX USA; 10 Texas Children’s Hospital, Houston, TX USA

## Abstract

**Background:**

In medical genetics, discovery and characterization of disease trait contributory genes and alleles depends on genetic reasoning, study design, and patient ascertainment; we suggest a segmental haploid genetics approach to enhance gene discovery and molecular diagnostics.

**Methods:**

We constructed a genome-wide map for nonallelic homologous recombination (NAHR)-mediated recurrent genomic deletions and used this map to estimate population frequencies of NAHR deletions based on large-scale population cohorts and region-specific studies. We calculated recessive disease carrier burden using high-quality pathogenic or likely pathogenic variants from ClinVar and gnomAD. We developed a NIRD (NAHR deletion Impact to Recessive Disease) score for recessive disorders by quantifying the contribution of NAHR deletion to the overall allele load that enumerated all pairwise combinations of disease-causing alleles; we used a Punnett square approach based on an assumption of random mating. Literature mining was conducted to identify all reported patients with defects in a gene with a high NIRD score; meta-analysis was performed on these patients to estimate the representation of NAHR deletions in recessive traits from contemporary human genomics studies. Retrospective analyses of extant clinical exome sequencing (cES) were performed for novel rare recessive disease trait gene and allele discovery from individuals with NAHR deletions.

**Results:**

We present novel genomic insights regarding the genome-wide impact of NAHR recurrent segmental variants on recessive disease burden; we demonstrate the utility of NAHR recurrent deletions to enhance discovery in the challenging context of autosomal recessive (AR) traits and biallelic variation. Computational results demonstrate new mutations mediated by NAHR, involving recurrent deletions at 30 genomic regions, likely drive recessive disease burden for over 74% of loci within these segmental deletions or at least 2% of loci genome-wide. Meta-analyses on 170 literature-reported patients implicate that NAHR deletions are depleted from the ascertained pool of AR trait alleles. Exome reanalysis of personal genomes from subjects harboring recurrent deletions uncovered new disease-contributing variants in genes including *COX10*, *ERCC6*, *PRRT2*, and *OTUD7A*.

**Conclusions:**

Our results demonstrate that genomic sequencing of personal genomes with NAHR deletions could dramatically improve allele and gene discovery and enhance clinical molecular diagnosis. Moreover, results suggest NAHR events could potentially enable human haploid genetic screens as an approach to experimental inquiry into disease biology.

**Supplementary Information:**

The online version contains supplementary material available at 10.1186/s13073-022-01113-y.

## Background

During the previous decade, efforts to decipher molecular and genetic mechanisms underlying Mendelian conditions have repeatedly demonstrated that mutations aggregate in personal genomes and can cause human diseases in a continuum of allelic modalities, ranging from monoallelic (dominant), biallelic (recessive), triallelic, to multiallelic and more complex modes of inheritance [1, 2]. Recent large-scale family-based genomic studies using exome sequencing (ES) have uncovered hundreds of new disease loci, with the majority following traditional Mendelian inheritance, i.e., monoallelic (autosomal dominant [AD]) or biallelic (autosomal recessive [AR]) trait segregation [3, 4]. Although optimism has been increasing towards achieving disease annotation for a substantial portion of the haploinsufficient part of the human genome through dominant disease gene discoveries, statistical analysis from rare disease cohort studies suggests that the trajectory to understanding, or illumination of the biology thereof, of the rare recessive disease traits, that is specifically the biallelic-disease-causing portions of the human genome, is less certain [5–7].

This apparent discrepancy in discovery between AD and AR trait genes can perhaps most parsimoniously be explained by the Clan Genomics Model [1]. The model predicts that dominant diseases are largely caused by emergence of new alleles in recent generations, i.e., de novo mutations (DNMs). On the other hand, recessive disease traits arise when a *pair* of disease alleles at a locus are aggregated within a personal genome in the *trans* configuration by transmission genetics, i.e., maternal + paternal haplotypes; the incidence of disease correlates with the product of the probabilities of sampling each of the two alleles, whether existing in a population or de novo, in a mating. Because of the bivalent nature of such alleles, the overall incidence of the recessive trait depends on the local allelic architecture, i.e., the snapshot of the frequency distribution of all pathogenic alleles [8]. This is a characteristic not observed in dominant disease traits, wherein the individual alleles act in solitude and the overall incidence mostly depends on new mutations [9].

Individual recessive trait alleles can emerge and be carried in populations, clans, and pedigrees with no impact on phenotype. Consequently, some recessive disease alleles can reach high population allele frequencies [e.g., 8×10^−4^ for NM_000520.6(*HEXA*):c.1274_1277dup (p.Tyr427fs), the most common Tay-Sachs disease allele] [10], which can be several orders of magnitude higher than alleles associated with dominant disease traits (~10^−8^ as an estimate for the de novo single-nucleotide variant rate) [11]. As one may surmise or expect, previous research efforts or clinical testing in unselected cohorts are likely to ascertain recessive trait diseases with at least one allele of high population frequency (because these individuals are relatively more prevalent in disease cohorts). In contrast, if a yet-to-be-defined recessive disease trait gene does not have appreciable pathogenic alleles represented at a sufficient population frequency, disease discovery and annotation of the gene would be greatly hampered due to the extremely low incidence and difficulty in ascertaining affected individuals, even when considering a worldwide population of 7.8 billion.

Special strategies and genetic and genomic approaches need to be implemented to overcome this potential “barrier to discovery” and characterization initiatives. Ascertaining patients in populations with an elevated coefficient of consanguinity and autozygosity is a widely applied and highly successful strategy for rare recessive trait disease gene discovery [12]. In this circumstance, the allele pool shrinks to the Clan of the patient’s extended family, which dramatically escalates the effective disease allele frequency compared to the baseline allele frequency in the general population [12, 13]. Thus, the probability of ascertaining patients with a recessive trait disorder increases considerably, because the sampling of the second allele occurs within the Clan rather than the general population [14]. Similarly, focusing on a specific geographic or ethnic population is another effective strategy, often attributed to available founder mutation alleles in the population studied [13].

Here, we present an alternative study design strategy to enhance the investigation of novel AR disease trait genes, and alleles in biallelic recessive traits, that are difficult to access by conventional methods. This strategy leverages loss-of-function (LoF) alleles caused by large recurrent genomic deletions rendering a locus haploinsufficient (for AD traits) or hemizygous (for AR traits). Recurrent genomic deletions are a subset of contiguous gene deletions that are characterized by a special type of mutational mechanism called nonallelic homologous recombination, or NAHR [15]. NAHR is mediated by ectopic recombination between highly similar repeat sequences termed low-copy repeats (LCRs) or segmental duplications (SDs) [16]. The human genome is evolutionarily structured to be highly enriched for SDs, which creates a large number of architectural hotspots for genomic disorders and mirror traits to emerge [17, 18].

Recurrent genomic deletions (or NAHR deletions) set up an ideal background for AR disease trait gene discovery due to a key property: they act as highly prevalent recessive alleles in comparison to other small variant, i.e., single-nucleotide variants (SNV) and indels, because of the injection of new mutations to maintain the allele load is persistently driven by the high mutation rates of structural variant mutagenesis. It has been shown that the mutation rate of NAHR at a given locus can be as high as ~10^−4^ to 10^−5^, which is orders of magnitudes higher than the per base new mutation rates from SNVs and indels [19]. The high new mutation rate ensures that these genomic deletions continuously arise de novo in the human population among unrelated individuals [20, 21]. This “recurrent” nature of genomic deletions distinguishes them from the other recessive trait disease alleles that are more “stationary” or ancestral artifacts of past population history amplified by recent population expansion. Moreover, as genotyping assays are performed on relatives of patients with deletions as well as on individuals without a disease indication, we recognize that many recurrent genomic deletions are incompletely penetrant [22–29], i.e., the fitness of the deletion allele can be high, at least in certain genomic backgrounds [30, 31].

Thus, we hypothesized that because of these key attributes, population prevalence and new mutation rate, NAHR alleles are contributing to a considerable fraction of recessive rare disease traits at loci mapped within NAHR genomic intervals and may be among the most relevant and prominent alleles at these recessive trait loci. Additionally, we hypothesize that although the sequencing of large recurrent deletions has resulted in isolated characterizations of new recessive genes and alleles in the past, such focused efforts have been under-recognized as a concerted generalizable study strategy, possibly due to the preconceived notion that most of the large recurrent deletions are “dominant” incompletely penetrant disease alleles.

**Fig. 1:**
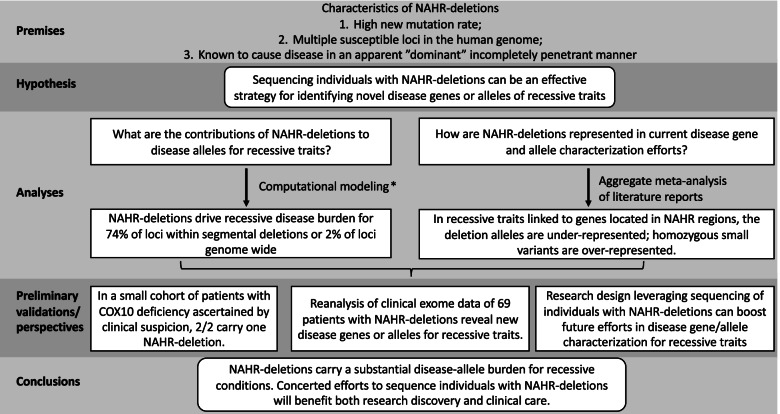
A flowchart of this study. Abbreviations: NAHR, nonallelic homologous recombination. *, a summary of the computational modeling is provided in Fig. 2

**Fig. 2:**
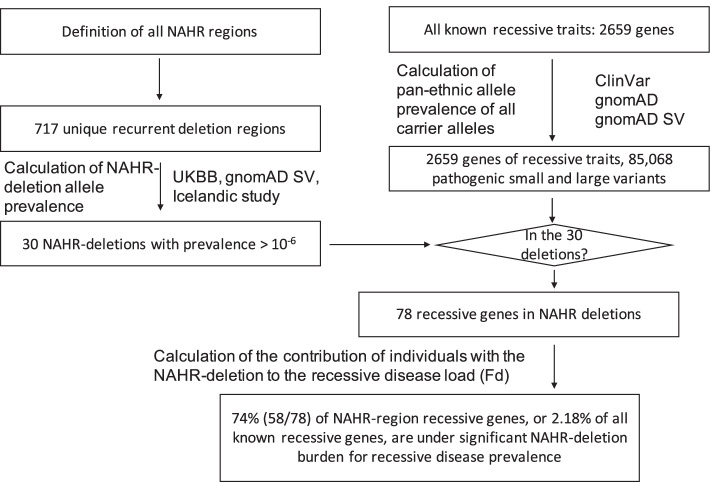
Computational modeling reveals unexpected contribution of NAHR deletions to autosomal recessive disease trait burden

To pursue these hypotheses, we formulated a large computational analysis using both genome-wide population data resources as well as clinical genomics empirically derived information (Fig. 1). Herein, we demonstrate, using computational and sequencing analyses, that recurrent NAHR deletions contribute to a major fraction of individual disease burden for over 2% of known recessive trait genes or 74% of known recessive disease traits in regions encompassed by LCRs, the latter genomic intervals known to undergo NAHR at elevated mutational rates [19, 20] (Fig. 2). By meta-analysis of all patients and disease alleles reported in the literature from the top rare recessive disease trait genes predicted to be driven by NAHR deletion alleles, we present evidence suggesting that the genomic sequencing of individuals with recurrent deletions is under-utilized. The findings regarding allelic architecture of diseases, leveraging new mutation and the high incidence of recurrent genomic deletion alleles, can “prime” powerful future strategies for recessive disease trait genes and allele discoveries exploiting the concept of “human haploid genetics,” from the original utilization of disease trait genes mapping on the X chromosome in affected males [32], to the current proposed application of investigating genomic intervals of recurrent segmental aneusomy for each of the diploid autosomal chromosomes.

## Methods

### Construction of a genome-wide map for NAHR-mediated recurrent genomic deletions

Possible loci for recurrent deletions were identified by enumerating all regions flanked by directly oriented low-copy repeat (LCR) pairs, LCR sometimes referred to as segmental duplication (SD) in the human genome. These LCR pairs stimulate NAHR-derived deletions of the genomic intervals mapping between the directly oriented pairs. A genomic interval containing the same set of genes can be flanked by different LCR pairs. LCR pairs clustering to the same NAHR region were computationally identified and reduced to generate the coordinates of the merged NAHR intervals.

Metrics that could inform estimation of the new mutation rates for each genomic disorder were kept for each pair of SD elements from the merged cluster of repeats, including repeat lengths, distance in between, and sequence similarity [33]. Gene content of the deleted segment is expected to influence the fitness of this allele. We used the number of genes with a high pLI score, i.e., greater intolerance to haploinsufficiency, to estimate the level of selection against the genomic deletion in the population. These metrics were used to calculate a score to inform relativization of the populational prevalence of these CNVs; the actual prevalence values used in the modeling of this study are obtained from empirical population or disease cohort studies as detailed below. Coordinates for deletion breakpoints are calculated as a weighted average of all the ranges of possible SDs that may mediate the deletion. Thus, the coordinates are not precise predictions for a specific deletion observed in individual patients, but rather average of all possible types of deletions that were collapsed into the merged deletion. Two separate NAHR deletion maps were generated using SegDup tracks from GRCh38 and GRCh37.

To account for the issue that relying on a reference human genome haplotype may lead to under-representation of recurrent deletion, we performed comprehensive literature review to search for recurrent deletions that only occur on alternative haplotypes. We found that the chromosome 17q21.31 recurrent deletion, which is known to occur on an alternative inversion haplotype [34, 35], is only represented in an alternative contig, chr17_GL000258v2_alt. Thus, this genomic region was manually patched to the analysis result as detailed in the GitHub code. We cannot exclude that additional recurrent deletion regions similar to the 17q21.31 deletion may be missed from this analysis if they are not well represented in the literature.

### Population cohorts used in this study

For genomic deletions with a population prevalence over 1/1,000,000, prevalence estimates were calculated based on the UK Biobank cohort [25], the Icelandic cohort [24], summary statistics from GeneReviews, gnomAD SV [23], or region-specific studies. The region-specific data sources used for the prevalence estimates are provided in Additional file 1: Table S1. The version of the UK Biobank cohort used in this study contained a sample size of 421,268, who all passed the genotyping and CNV calling QC described in Crawford et al. [25]. The participants were recruited from the general population of the UK, using National Health Service patient registers, with no exclusion criteria. They aged between 40 and 69 years at the time of recruitment (2006 to 2010). The samples were analyzed by the Affymetrix UK BiLEVE Array (807,411 probes) or the Affymetrix UK Biobank Axiom Array (820,967 probes), and the CNVs were called by the PennCNV-Affy software [25]. The Icelandic cohort consists of 101,655 subjects aged 18 to 65 years, representing approximately one-third of the Icelandic population [24]. According to Stefansson et al., the sample had been genotyped by Illumina HumanHap (300, 370, 610, 1M, 2.5M) and Illumina Omni (670, 1M, 2.5M, Express) SNP arrays, and CNV calls were performed by PennCNV [24]. The gnomAD cohort consists of 14,237 adult individuals (median age of 49 years); CNV calling was performed on the WGS data using a cloud-based, multi-algorithm pipeline for short-read WGS [23].

### Prevalence curation for NAHR-mediated recurrent genomic deletions

First, prevalence estimates from the two largest and the most systematic datasets, the UK Biobank and the Icelandic cohort, are compared. If the prevalence estimates were not significantly different (Fisher’s exact test, *p*>0.05), the UK Biobank prevalence was taken since this study has the largest cohort size. Otherwise, prevalence from a region-specific cohort was compared with estimates from the UK Biobank and the Icelandic cohorts, and the group with a closer match was taken.

Then, we queried each deletion region in GeneReviews for reputable prevalence estimates. When the GeneReviews prevalence differs significantly from the UK Biobank and the Icelandic cohort provided numbers, we further manually investigate the literature to determine which study may be the most appropriate to be used.

For genomic deletions with a prevalence lower than 1/1,000,000, we investigated a cohort of 33,452 patients who were referred for clinical chromosomal microarray analysis (CMA) using custom designed Agilent oligo-based comparative genomic hybridization arrays [36]. Of note, deletion prevalence estimates from the CMA cohort do not represent actual prevalences in the general population, but can inform relative prevalence comparison among rare variant mutational events in the population.

### Recessive disease carrier burden calculation

High-quality ClinVar variants were defined as having a pathogenic or likely pathogenic label with at least one-star review status (accessed 01/21/2021). LoF variants from gnomAD SV were defined by variants meeting all the following criteria (1) PASS filter in the VCF file with a quality score over 500, (2) PROTEIN_CODING__LOF flag or PROTEIN_CODING__DUP_LOF flag in the VCF file, (3) POPMAX allele frequency lower than 1% and no homozygote counts, (4) less than 80% of the span overlapping with segmental duplications, and (6) the LoF consequence affects all RefSeq transcripts of a recessive gene.

High-confidence LoF small variants from gnomAD v3.1 were defined by variants that fulfill all the following criteria: (1) PASS filter from the gnomAD v3.1 VCF file, (2) do not fall into a low complexity region, (3) QUALapprox score lower than 1×10^5^, (4) sequenced in over 7.5×10^4^ alleles, (5) population allele frequency lower than 1% with no homozygous counts, and (6) marked as a high-quality LoF variant by LOFTEE [37].

### Calculation of NAHR deletion contribution to disease burden for a specific recessive disorder

All the following calculations are based on the concept of random mating by sampling from a pool of alleles. Suppose that at an autosomal recessive trait locus, we have *n* alleles, *A*_1_, *A*_2_, …, *A*_k_, …, *A*_n_, with allele frequencies *p*_*1*_, *p*_*2*_, …, *p*_*k*_, …, *p*_*n*_. Without loss of generality, we nominate the *k*th allele as the NAHR deletion allele—our allele of interest—and let the others index the small variant alleles. For most large recurrent deletion CNVs, homozygous loss of the deletion is incompatible with live birth.

The two exceptions are the 2q13-*NPHP1* deletion and the 15q13.3 BP4-BP5 deletion, for which homozygous deletions are compatible with live birth. Also, the 15q13.3 BP4-BP5 deletion is encompassed by the 15q11q13 BP3-BP5 deletion, so the enclosed recessive genes have two NAHRdelCNV alleles contributing to them.

Hypomorphic variant alleles that cause disease when they co-occur in combination with LoF alleles, which we denote as A_h_, are not disease-causing in the homozygous states. Such alleles are observed in *RBM8A* and *TBX6*, but at present not in others. The exceptions described above regarding *RBM8A*, *TBX6*, the 2q13-*NPHP1* deletion, and the 15q13.3 BP4-BP5 deletion have been accounted for in the calculations for these special circumstances. For simplicity, they are not incorporated in the equations below for illustration, although the modifications to account for them are simple adjustments to the sums and formulas presented below. The modified equations used in the modeling are described in the supplementary methods (Additional file 2: Supplementary Methods) as well as reflected in the online code.

We denote the probability of an individual carrying the NAHR deletion allele is

$$P\left({A}_k\right)={p}_k$$

The contribution of the NAHR deletion to the allele load (**Fa**) is the fraction of the NAHR deletion allele frequency over the sum of all allele frequencies across all functional alleles—except for the hypomophric alleles that do not in themselves cause disease.

$$F{a}_k=\frac{p_k}{\sum_{i=1}^n{p}_i}$$

The Punnett square below models the expected frequencies of an individual to be affected with the recessive trait given the population frequencies of each pair of alleles under a random mating model.

**Table:** 

	*A*_1_*p*_*1*_	*A*_2_*p*_*2*_	…	*A*_k_*p*_*k*_	**…**	*A*_n_*p*_*n*_
*A*_1_ *p*_*1*_	***p***_***1***_^**2**^ , ***A***_**1**_***A***_**1**_	***p***_***1***_***p***_***2***_ , ***A***_**1**_***A***_**2**_	**…**	***p***_***1***_***p***_***k***_ , ***A***_**1**_***A***_**k**_	**…**	***p***_***1***_***p***_***n***_ , ***A***_**1**_***A***_**n**_
*A*_2_ *p*_*2*_	***p***_***1***_***p***_***2***_ , ***A***_**1**_***A***_**2**_	***p***_***2***_^**2**^ , ***A***_**2**_***A***_**2**_	**…**	***p***_***2***_***p***_***k***_ , ***A***_**2**_***A***_**k**_	**…**	***p***_***2***_***p***_***n***_ , ***A***_**2**_***A***_**n**_
⁝	⁝	⁝	**…**	**…**	**…**	**…**
*A*_k_ *p*_*k*_	***p***_***1***_***p***_***k***_ , ***A***_**1**_***A***_**k**_	***p***_***2***_***p***_***k***_ , ***A***_**2**_***A***_**k**_	**…**	***p***_***k***_^**2**^ , ***A***_**k**_***A***_**k**_	**…**	***p***_***k***_***p***_***n***_ , ***A***_**k**_***A***_**n**_
⁝	⁝	⁝	**…**	⁝	**…**	***…***
*A*_n_ *p*_*n*_	***p***_***1***_***p***_***n***_ , ***A***_**1**_***A***_**n**_	***p***_***2***_***p***_***n***_ , ***A***_**2**_***A***_**n**_	**…**	***p***_***k***_***p***_***n***_ , ***A***_**k**_***A***_**n**_	***…***	***p***_***n***_^**2**^ , ***A***_**n**_***A***_**n**_

The probability for an individual to be affected with the recessive disorder is the sum of pairwise products of all carrier alleles with contribution from the homozygous NAHR deletion lethal allele subtracted

$$P(D)=\sum_{i=1}^n\sum_{j=1}^n{p}_i{p}_j-{p}_k^2$$

The probability for an individual to be both affected with the recessive disorder and carrying the NAHR deletion is

$$P\left({A}_k\cap D\right)=2\bullet \sum_{j=1}^n{p}_k{p}_j-2\bullet {p}_k^2$$

The contribution of individuals with the NAHR deletion to the recessive disease load (**Fd**) is

$${Fd}_k=P\left({A}_k\left|D\right.\right)=\frac{P\left({A}_k\cap D\right)}{P(D)}$$

The odds for an individual with the recessive disease to carry the NAHR deletion is

$${O}_k=\frac{Fd_k}{\sum_{i=1}^n{Fd}_i-{Fd}_k}$$

Note that the sum of Fd across alleles *1*, *2*, *3*, …, *n* equals the sum of the Punnett square matrix plus the lower triangular and the upper triangular. This is equivalent to summing up homozygous allele products once plus compound heterozygous allele products twice. This characteristic arises because the events of recessive disease involving the *k*th and the *j*th allele are not disjoint and they overlap for the compound heterozygote entries in the Punnett square.

To calculate the NAHR deletion Impact to Recessive Disease score, the odds of the NAHR allele is compared to that of the “median” allele from the same gene.

$$NIRD={\log}_2\left({OR}_k\right)={\log}_2\left(\frac{O_k}{O_{median}}\right)$$

The “median” allele is defined as the midpoint of remaining alleles in the same gene that comprise a cumulative sum of the top 90% of the overall sum of Fd. The alleles consisting the lower 10% of overall Fd sum are disregarded because (1) we find many genes have a long tail of ultra-rare alleles without a frequency estimate from gnomAD, and (2) we supplemented 10% of hypothetical alleles to each gene in our analysis.

### Curation of recessive disease trait alleles from the meta-analysis of the 170 patients identified from the literature

The Human Gene Mutation Database (HGMD, version 2020.4) [38] was queried for recessive disease trait alleles in genes for which ≥70% of the disease carrier burden was attributed to NAHR-mediated large deletions; i.e., NAHRdelCNV. The literature linked to each disease allele that was marked as a disease-causing mutation (DM) or possible DM (DM?) was mined for the allelic state of the variant (homozygous, compound heterozygous with another SNV, or compound heterozygous with the NAHR-mediated deletion, NAHRdelCNV) as well as the number of unrelated families carrying the allele. Homozygous variants were counted once per family, while compound heterozygous SNVs received one count each per family. Gross deletions and insertions larger than 50 bp were excluded. *RBM8A* was the only gene for which disease-associated polymorphisms, with additional supporting functional evidence (DFP), were also included; DM/DM? alleles as compound heterozygous with DFP variants were dismissed for all genes other than *RBM8A*. *MYH11* and *PMP22* are the only two genes in this curation effort to have disease associations of both AD trait and AR trait inheritance. *MYH11* alleles were only added if they were associated with hypoperistalsis. Similarly, only biallelic loss-of-function *PMP22* alleles were included. The total population frequency of each curated allele was retrieved using gnomAD v3.1.

### Retrospective analyses of extant cES for a novel rare recessive disease trait gene and allele discovery from individuals with recurrent genomic deletions

The patients were evaluated by clinical exome sequencing (cES), with sequencing, data analysis, and interpretation procedures described previously [39, 40]. The identification of deletion CNVs was based on a SNP array platform, which was performed concurrently with the exome assay [41]. Sanger dideoxy DNA sequencing was performed as a validation method for candidate diagnostic small variant alleles.

## Results

### NAHR deletion: the most prevalent disease allele for a major fraction of recessive trait genes mapping to 30 genomic loci

In order to systematically evaluate the contribution of recurrent genomic deletions to autosomal recessive conditions, we first mapped all possible loci that are susceptible to recurrent deletions caused by NAHR between directly oriented SDs [42, 43] using the GRCh38 human reference genome sequence (Additional file 3: Figure S1, Additional file 1: Table S2). The collapsed NAHR map contains 717 unique recurrent deletion regions. We enumerated the subset of recurrent deletion events with available data from screening efforts in the literature or clinical testing to substantiate a prevalence estimate, and focused the subsequent analyses on these genomic intervals (*n*=51).

We identified 30 autosomal deletions with a maximal population prevalence over 1/1,000,000 based upon estimates from the UK Biobank, the Icelandic, the gnomAD SV database, or region-specific studies [23–25] (Table 1, Additional file 1: Table S1). Of note, these deletion allele frequencies reflect empirical prevalence measurements from adult populations, which closely represent the effective allele frequencies (i.e., combined consideration of both the de novo mutation rate and fitness of the variant on a cellular, developmental, and organismal level) suited for recessive disease trait load estimation. These 30 deletions span 64 Mb of unique genomic sequences in the assayable portion of the human genome, contribute to an aggregate population allele burden of 1.3%, and encompass 1555 genes, of which 78 are known to cause recessive disorders. An additional 20 deletions, with populational prevalence possibly lower than 1/1,000,000, are also identified to recur in high prevalence if a clinical cohort is ascertained (Additional file 1: Table S1). With the 20 ultra-rare deletions included, the span of genomic coverage increases to 82 Mb; the number of genes involved becomes 1875, with 101 representing established recessive disease trait genes. Moreover, the “haploid genetics” concept begins to emerge as an approach based on observational data and data analyses.

**Table 1:** Recurrent genomic deletions that are prevalent in the population

Region	Coordinates (GRCh38)	Population allele frequency (× 10^−6^)	Allele frequency in diagnostic testing (× 10^−6^)	Known recessive genes	Number of coding genes
2q13 *NPHP1*	chr2:109930242-110228182	5811	2616	*NPHP1*	3
15q11.2	chr15:21311962-23261294	2764	2287	*-*	14
16p12.1	chr16:21754781-22502804	584.0	627.8	*OTOA*, *UQCRC2*	11
16p11.2 proximal	chr16:29416551-30202090	507.6	1674	*PRRT2*, *ALDOA*, *TBX6*, *CORO1A*	35
17p12 HNPP	chr17:14170711-15567588	314.8	388.6	*COX10*, *PMP22*	9
16p13.11	chr16:14772948-16330433	311.0	433.5	*NDE1*, *MYH11*, *ABCC6*	15
1q21.1 BP3-BP4	chr1:146380249-148811725	268.2	672.6	*-*	15
13q12.12	chr13:22911590-24323812	201.8	104.6	*SGCG*, *SACS*, *MIPEP*	7
1q21 TAR	chr1:144904297-146209950	178.0	269.0	*PEX11B*, *RBM8A*, *POLR3GL*, *HJV*	22
22q11.2 LCRA-D	chr22:18530098-21214537	141.7	4499	*PRODH*, *SLC25A1*, *CDC45*, *GP1BB*, *TXNRD2*, *TANGO2*, *SCARF2*, *PI4KA*, *SNAP29*, *LZTR1*	48
10q11.21q11.23	chr10:45765081-49954967	135.3	74.73	*RBP3*, *ERCC6*, *SLC18A3*, *CHAT*	38
16p11.2 distal	chr16:28706949-29049993	137.7	254.1	*TUFM*, *ATP2A1*, *CD19*, *LAT*	11
2q13	chr2:110494056-112385043	125.8	149.5	*ANAPC1*, *MERTK*	11
7q11.23	chr7:73089294-74862006	120.0	1375	*NCF1*	28
2q21.1	chr2:130623447-131386379	97.33	119.6	*-*	9
15q13.3 BP4-BP5	chr15:30246847-32496522	99.70	896.8	*FAN1*, *TRPM1, OTUD7A*	13
2q11.2	chr2:95759114-97430329	73.60	74.73	*ADRA2B*, *NCAPH*, *LMAN2L*, *CNNM4*	24
17q12	chr17:36300613-38034442	68.86	463.4	*ZNHIT3*, *PIGW*	21
17p11.2 Smith Magenis Syndrome	chr17:16777950-20450859	53.33	687.6	*TNFRSF13B*, *ATPAF2*, *MYO15A*, *MEIF2*, *TOP3A*, *GRAP*, *B9D1*, *ALDH3A2*	48
15q11q13 BP3-BP4	chr15:28580349-30417865	37.98	134.5	*NSMCE3*	10
3q29	chr3:195963652-197626678	21.36	194.3	*TFRC*, *PCYT1A*, *TCTEX1D2*, *RNF168*, *NRROS*, *CEP19*	23
17q11.2	chr17:30621877-32037969	21.36	149.5	*-*	14
15q11 Prader-Willi/ Angelman syndromes BP1-BP3	chr15:21976318-28537425	19.89	687.6	*OCA2*, *HERC2*	27
15q11 Prader-Willi/ Angelman syndromes BP2-BP3	chr15:23247414-28447477	19.89	657.7	*OCA2*, *HERC2*	17
22q11.2 LCRD-H	chr22:21206521-24255497	11.87	59.79	*IGLL1*	45
8p23.1	chr8:7596999-12344083	9.495	134.5	*RP1L1*, *FDFT1*	50
10q23	chr10:79733715-87254783	7.121	59.79	*MAT1A*, *CDHR1*	31
15q24 BPA-BPC	chr15:72628218-75278711	2.374	74.73	*BBS4*, *STRA6*, *EDC3*, *MPI*, *COX5A*	40
15q11q13 BP3-BP5	chr15:28569118-32447357	2.374	59.79	*NSMCE3*, *FAN1*, *TRPM1, OTUD7A*	21
7q11.23 distal	chr7:75456184-76629927	2.374	29.89	*POR*, *MDH2*	19

Regions are listed in descending order by population prevalence. Genes in the “Known recessive gene” column are ordered by coordinate map positions. Even though it is the third highest NAHR-mediated deletions, the Xp22.31-*STS* deletion is not included in this table because the current list focuses on autosomal recessive conditions. The gene *OTUD7A* in 15q13.3 BP4-BP5 is not reported to cause a recessive disease at the time of this study; however, patient analysis results from this study support this gene as a candidate recessive disease gene

We then catalogued, based on existing knowledge and datasets, a compendium of all reported and predicted carrier alleles for each known recessive trait gene in the human genome. Our objective for this recessive allele catalog is to estimate and dissect the impact of the new mutation recurrent genomic deletions’ contribution to the overall disease burden. Based on mode of inheritance curations from OMIM [44], DECIPHER [45], and ClinGen [46] (data accessed on 1/4/2021), a totality of 2659 recessive disease trait genes were assembled. The carrier allele burden for each “recessive trait gene” was calculated by summing up frequencies of unique alleles for all high-quality pathogenic variants from ClinVar, all structural variants (SV) predicted to be LoF from gnomAD SV v2.1, all high-confidence LoF small variants identified in gnomAD v3.1, and the NAHR-mediated recurrent genomic deletions, if applicable. An aggregate of 85,068 small variant and large deletion carrier alleles were identified for the 2659 genes (Additional file 1: Table S3). For the 78 known recessive genes in the NAHR deletion regions, the number of per gene pathogenic alleles range from 1 to 308, with a median of 14. As a comparison, the remaining 2580 known rare recessive disease trait genes have a similar median per gene pathogenic allele count, 14, but a wider range, from 1 to 3562.

A limitation of this calculation is that SNV pathogenic missense, in-frame indel, or intronic variants not currently reported in ClinVar are inadvertently omitted. However, we argue that carrier alleles not represented in ClinVar tend to have lower allele frequencies and thus do not have a major impact on the subsequent carrier burden estimates. We further argue that the alleles that receive an entry and curation in ClinVar have higher frequencies—and therefore greater impact on recessive disease, and these are the alleles more easily ascertained in screening tests of clinical diagnostic laboratories. This latter contention is supported by the aggregate gene-level carrier allele burden from our analysis matching empirical experience in genetic testing carrier screenings results (Additional file 1: Table S4) [47].

Nevertheless, to account for potential unrepresented alleles from recessive disease trait genes that have not been scrutinized by large-scale systematic clinical or research screening, in the subsequent analyses, we supplemented the disease allele pool for each gene with a 10% extra variant load, comprised of ten hypothetical variants each accounting for 1% of the overall carrier burden (see “Methods”) for each gene. Of note, the NAHR deletion alleles rank as the most (49/78) or second most (11/78) frequent (highest population allele frequency) carrier alleles together comprising over three quarters of known recessive trait genes within NAHR regions! Even with the abovementioned conservative “padding” to represent ten hypothetical alleles not yet ascertained, the NAHR alleles still contribute to greater than 10% of the total gene-level carrier allele burden for 60 of the 78 genes (Additional file 1: Table S5).

### NAHR deletions contribute a major fraction of recessive disease load to genes mapping within rearrangement hotspots

It is important to note that, for a recessive trait, the population frequency and relativized frequency of a particular allele from a pool of alleles (**Fa**, fraction of allele burden) is not linearly correlated with the probability of sampling a patient with the specific allele from a group of patients (**Fd**, fraction of the disease burden). The distribution of alleles in affected individuals is determined by the pairwise allele frequency products in a pool.

Thus, we calculated allelic contributions to recessive disease load using an *n* × *n* Punnett square, where *n* is the number of carrier alleles for a recessive disease trait gene. The calculated NAHR deletion contribution to disease can be calculated from the matrix. We denote Fd as the modeled probability of sampling individual carrying at least one recurrent deletion allele from a pool of patients affected with the recessive condition caused by the same gene. We empirically considered a gene to be under significant NAHR deletion burden for population prevalence of the associated recessive disease trait, if the recurrent genomic deletion is expected in greater than 20% of all patients with this recessive disorder. By this definition, 74% (58/78) of NAHR-region recessive genes, which account for 2.184% of all known recessive genes, are under significant NAHR deletion burden for recessive disease trait prevalence (Table 2)! In the context of the other alleles from the same gene, the disease contribution of the NAHR deletion (Fd) ranks at the top for 49 genes, and at second place for 11 more genes. The Fd scores of the top 3 alleles are listed in Table 2 and Additional file 1: Table S5 to illustrate a snapshot of the allelic architecture for each recessive trait gene.

**Table 2:** Recessive genes with NAHR-mediated recurrent genomic deletions contributing to more than 20% of the overall disease burden

Gene	AR disease trait	Genomic region cytogenetic interval	NAHR deletion prevalence in adults	***N***AHR deletion ***I***mpact to ***R***ecessive ***D***isease (NIRD)	Top 3 allele contribution to disease^a^ (NAHRdelCNV in bold)	Fraction of NAHRdelCNV allele frequency	Aggregate carrier allele frequency
*NPHP1*	Nephronophthisis 1, juvenile, MIM# 256100	2q13 *NPHP1*	5.811x10^-3^	7.7	**98**, 3, 2	85	6.82x10^-3^
*ADRA2B*	Autosomal recessive mental retardation (from DECIPHER)	2q11.2	7.36x10^-5^	4.1	**95**, 10, 10	90	8.18x10^-5^
*ALDOA*	Glycogen storage disease XII, MIM# 611881	16p11.2 proximal	5.076x10^-4^	4.1	**95**, 10, 10	90	5.64x10^-4^
*CEP19*	Morbid obesity and spermatogenic failure, MIM# 615703	3q29	2.136x10^-5^	4.1	**95**, 10, 10	90	2.37x10^-5^
*CORO1A*	Immunodeficiency 8, MIM# 615401	16p11.2 proximal	5.076x10^-4^	4.1	**95**, 10, 10	90	5.64x10^-4^
*COX5A*	Mitochondrial complex IV deficiency, nuclear type 20, MIM# 619064	15q24 BPA-BPC	2.374x10^-6^	4.1	**95**, 10, 10	90	2.64x10^-6^
*EDC3*	Mental retardation, autosomal recessive 50, MIM# 616460	15q24 BPA-BPC	2.374x10^-6^	4.1	**95**, 10, 10	90	2.64x10^-6^
*LAT*	Immunodeficiency 52, MIM# 617514	16p11.2 distal	1.377x10^-4^	4.1	**95**, 10, 10	90	1.53x10^-4^
*MIEF2*	Combined oxidative phosphorylation deficiency 49, MIM# 619024	Smith Magenis Syndrome	5.333x10^-5^	4.1	**95**, 10, 10	90	5.93x10^-5^
*SLC18A3*	Myasthenic syndrome, congenital, 21, presynaptic, MIM# 617239	10q11.21q11.23	1.353x10^-4^	4.1	**95**, 10, 10	90	1.50x10^-4^
*PMP22*	Dejerine-Sottas disease, MIM# 145900	17p12 HNPP	3.148x10^-4^	4.2	**94**, 11, 9	89	3.54x10^-4^
*OTUD7A*	Neurodevelopmental disorder (this study)	15q13.3 BP4-BP5	9.97x10^-5^	5.4	**93**, 10, 9	74	1.35x10^-4^
*GRAP*	Deafness, autosomal recessive 114, MIM# 618456	Smith Magenis Syndrome	5.333x10^-5^	4.6	**91**, 41, 7	84	6.38x10^-5^
*RBM8A*	Thrombocytopenia-absent radius syndrome, MIM# 274000	1q21.1 TAR	1.78x10^-4^	9.8	**90**, 1, 1	90	1.98x10^-4^
*PRODH*	Hyperprolinemia, type I, MIM# 239500	22q11.2 LCRA-D	1.417x10^-4^	5	**85**, 39, 28	75	1.90x10^-4^
*UQCRC2*	Mitochondrial complex III deficiency, nuclear type 5, MIM# 615160	16p12.1	5.84x10^-4^	5	**85**, 50, 7	75	7.83x10^-4^
*NDE1*	Lissencephaly 4 (with microcephaly), MIM# 614019	16p13.11	3.11x10^-4^	5.1	**84**, 54, 15	72	4.30x10^-4^
*PEX11B*	Peroxisome biogenesis disorder 14B, MIM# 614920	1q21 TAR	1.78x10^-4^	4.7	**83**, 21, 18	72	2.48x10^-4^
*PRRT2*	Autosomal recessive mental retardation (from DECIPHER)	16p11.2 proximal	5.076x10^-4^	5.2	**79**, 60, 8	65	7.77x10^-4^
*TUFM*	Combined oxidative phosphorylation deficiency 4, MIM# 610678	16p11.2 distal	1.377x10^-4^	3.9	**79**, 10, 10	65	2.13x10^-4^
*COX10*	Mitochondrial complex IV deficiency, nuclear type 3, MIM# 619046	17p12 HNPP	3.148x10^-4^	5.1	**77**, 48, 25	62	5.05x10^-4^
*POLR3GL*	Endosteal Hyperostosis and Oligodontia (from DECIPHER)	1q21 TAR	1.78Ex10^-4^	4.1	**74**, 20, 13	59	3.00x10^-4^
*SCARF2*	Van den Ende-Gupta syndrome, MIM# 600920	22q11.2 LCRA-D	1.417x10^-4^	3.8	**73**, 38, 8	58	2.45x10^-4^
*B9D1*	Joubert syndrome 27, MIM# 617120	Smith Magenis Syndrome	5.333x10^-5^	5.3	**72**, 74, 12	56	9.47x10^-5^
*MYH11*	Megacystis-microcolon-intestinal hypoperistalsis syndrome (from DECIPHER)	16p13.11	3.11x10^-4^	5	**72**, 24, 7	57	5.48x10^-4^
*NCAPH*	Microcephaly 23, primary, autosomal recessive, MIM# 617985	2q11.2	7.36x10^-5^	2.9	**71**, 27, 27	55	1.33x10^-4^
*ANAPC1*	Rothmund-Thomson syndrome, type 1, MIM# 618625	2q13	1.258x10^-4^	3.8	**66**, 13, 11	50	2.54x10^-4^
*CD19*	Immunodeficiency, common variable, 3, MIM# 613493	16p11.2 distal	1.377x10^-4^	4	**65**, 38, 28	48	2.86x10^-4^
*HJV*	Hemochromatosis, type 2A, MIM# 602390	1q21 TAR	1.78x10^-4^	5.2	**62**, 83, 5	45	3.97x10^4^
*PIGW*	Glycosylphosphatidylinositol biosynthesis defect 11, MIM# 616025	17q12	6.886x10^-4^	5.2	**61**, 64, 35	44	1.56x10^-4^
*IGLL1*	Agammaglobulinemia 2, MIM# 613500	22q11.2 LCRD-H	1.187x10^-5^	5.2	**61**, 51, 51	43	2.79x10^-5^
*ATPAF2*	Mitochondrial complex V (ATP synthase) deficiency, nuclear type 1, MIM# 604273	Smith Magenis Syndrome	5.333x10^-5^	4.1	**58**, 78, 12	41	1.29x10^-4^
*CNNM4*	Jalili syndrome, MIM# 217080	2q11.2	7.36x10^-5^	3.2	**57**, 16, 9	40	1.84x10^-4^
*RBP3*	Retinitis pigmentosa 66, MIM# 615233	10q11.21q11.23	1.353x10^-4^	4.1	**58**, 30, 9	40	3.42x10^-4^
*SLC25A1*	Myasthenic syndrome, congenital, 23, presynaptic, MIM# 618197	22q11.2 LCRA-D	1.417x10^-4^	4.2	**56**, 40, 16	38	3.69x10^-4^
*FDFT1*	Squalene synthase deficiency, MIM# 618156	8p23.1	9.495x10^-6^	5.1	**55**, 53, 53	38	2.52x10^-5^
*GP1BB*	Giant platelet disorder, isolated, MIM# 231200	22q11.2 LCRA-D	1.417x10^-4^	4.6	86, **55**, 5	38	3.75x10^-4^
*SGCG*	Muscular dystrophy, limb-girdle, autosomal recessive 5, MIM# 253700	13q12.12	2.018x10^-4^	4.7	**53**, 25, 16	36	5.62x10^-4^
*CHAT*	Myasthenic syndrome, congenital, 6, presynaptic, MIM# 254210	10q11.21q11.23	1.353x10^-4^	4.1	**52**, 25, 7	35	3.92x10^-4^
*SNAP29*	Cerebral dysgenesis, neuropathy, ichthyosis, and palmoplantar keratoderma syndrome, MIM# 609528	22q11.2 LCRA-D	1.417x10^-4^	4.3	**50**, 46, 29	33	4.23x10^-4^
*CDC45*	Meier-Gorlin syndrome 7, MIM# 617063	22q11.2_LCRA-D	1.417x10^-4^	4.2	**49**, 31, 20	33	4.34x10^-4^
*TBX6*	Spondylocostal dysostosis 5, MIM# 122600	16p11.2 proximal	5.076x10^-4^	5.6	**47**, 20, 5	47	1.08x10^-3^
*NSMCE3*	Lung disease, immunodeficiency, and chromosome breakage syndrome, MIM# 617241	15q11q13 BP3-BP4	3.798x10^-5^	4.8	91, **46**, 4	29	1.29x10^-4^
*NRROS*	Seizures, early-onset, with neurodegeneration and brain calcification, MIM# 618875	3q29	2.136x10^-5^	4.8	93, **44**, 2	29	7.48x10^-5^
*TFRC*	Immunodeficiency 46, MIM# 616740	3q29	2.136x10^-5^	1.6	**39**, 42, 29	24	8.94x10^-5^
*PCYT1A*	Spondylometaphyseal dysplasia with cone-rod dystrophy, MIM# 608940	3q29	2.136x10^-5^	1.8	**37**, 39, 17	22	9.49x10^-5^
*SACS*	Spastic ataxia, Charlevoix-Saguenay type, MIM# 270550	13q12.12	2.018x10^-4^	4.4	**36**, 14, 13	22	9.09x10^-4^
*OTOA*	Deafness, autosomal recessive 22, MIM# 607039	16p12.1	5.84x10^-4^	4.4	80, **35**, 5	21	2.78x10^-3^
*LMAN2L*	Mental retardation, autosomal recessive, 52, MIM# 616887	2q11.2	7.36x10^-5^	3.4	**32**, 32, 26	19	3.89x10^-4^
*MIPEP*	Combined oxidative phosphorylation deficiency 31, MIM# 617228	13q12.12	2.018x10^-4^	3.9	**32**, 15, 14	19	1.05x10^-3^
*TXNRD2*	Glucocorticoid deficiency 5, MIM# 617825	22q11.2 LCRA-D	1.417x10^-4^	3.4	49, 37, **31**	18	7.86x10^-4^
*ATP2A1*	Brody myopathy, MIM# 601003	16p11.2 distal	1.377x10^-4^	4.1	73, **30**, 7	18	7.79x10^-4^
*PI4KA*	Polymicrogyria, perisylvian, with cerebellar hypoplasia and arthrogryposis, MIM# 616531	22q11.2 LCRA-D	1.417x10^-4^	4.3	**28**, 16, 14	16	8.68x10^-4^
*DYNLT2B*	Short-rib thoracic dysplasia 17 with or without polydactyly, MIM# 617405	3q29	2.136x10^-5^	1.1	60, 44, **27**	16	1.36x10^-4^
*TANGO2*	Metabolic encephalomyopathic crises, recurrent, with rhabdomyolysis, cardiac arrhythmias, and neurodegeneration, MIM# 616878	22q11.2 LCRA-D	1.417x10^-4^	2.6	80, **23**, 23	13	1.11x10^-3^
*ZNHIT3*	PEHO syndrome, MIM# 260565	17q12	6.886x10^-5^	3.7	96, **22**, 2	13	5.47x10^-4^
*ERCC6*	Cockayne syndrome, type B, MIM# 133540	10q11.21q11.23	1.353x10^-4^	3.5	**21**, 15, 14	12	1.17x10^-3^
*FAN1*	Interstitial nephritis, karyomegalic, MIM# 614817	15q13.3 BP4-BP5	9.97x10^-5^	3.0	23, **21**, 20	11	8.94x10^-4^
*ALDH3A2*	Sjogren-Larsson syndrome, MIM# 270200	Smith Magenis Syndrome	5.333x10^-5^	2.7	41, 24, **20**	11	4.71x10^-4^

^a^The rows of the table are arranged in a descending order based on the NAHR deletion allele’s contribution to the recessive disease, i.e., the percent of all affected individuals carrying at least one NAHR deletion allele, which are represented as the bold numbers in the column “Top 3 allele contribution to disease.” Note that the numbers from the column “Top 3 allele” are not disjunct events. For example, a patient can be compound heterozygous for the top 1 allele and the 2nd allele, and thus contribute to both numbers. Genes with an NAHR allele contribution to disease lower than 20% are not tabulated here, but can be found in Additional file 1: Table S5

We next defined a log-scaled index we termed the NAHR deletion’s Impact to Recessive Disease (NIRD), to depict the gene-level disease load contribution of the NAHR allele relative to an allele with a median level of contribution to the same gene among all population carrier alleles (See “Methods” section). A positive NIRD score predicts that the NAHR deletion allele plays a predominant (above the typical allele) role among all carrier alleles of the gene in disease contribution, whereas a negative score predicts a minor (below typical) role. Known recessive genes in the recurrent deletion region tend to have high NIRD scores, with 91% (71/78) scoring above 0, and 79% (62/78) scoring above 2. Of note, the two highest NIRD scores are found in *RBM8A* and *NPHP1*, 9.8 and 7.7, respectively. Both are extremely large values considering the NIRD is log-scaled.

To appreciate the properties of the NIRD scores, we adjusted the algorithm to calculate the disease contribution of any given pathogenic allele for a recessive trait gene, as an Allelic Impact to Recessive Disease (AIRD) score. The most common carrier allele observed in cystic fibrosis, NM_000492.3(*CFTR*):c.1521_1523delCTT (p.Phe508delPhe), also known as the ΔF508 allele, has an AIRD score of 7.3; the third most common carrier allele for Niemann-Pick disease type A, NM_000543.5(*SMPD1*):c.996del (p.Phe333fs), has an AIRD score of 2; a well-known founder mutation observed in ~10% of patients of Ashkenazi Jewish descent with Tay-Sachs disease, NM_000520.6(*HEXA*):c.1421+1G>C, has an AIRD score of −0.15, due to its lower allele frequency of 1.97 × 10^−5^ in the general population according to gnomAD v3.1.

The NIRD and related findings provide the computational framework that supports two consequences. First, for the ~2% of known human recessive genes genome-wide or 74% of recessive genes in NAHR regions, one of the most effective but under-utilized approaches and strategies for identifying novel disease-causing alleles from human subjects for these genes is to sequence affected individuals carrying the heterozygous recurrent genomic deletion encompassing the gene of interest. Second, there likely exist uncharacterized recessive disease trait genes that may be most effectively identified by sequencing individuals bearing prevalent recurrent genomic deletions—i.e., any of the remaining 1477 genes within these deletion regions may have yet to be assigned an AR disease trait and could be novel biallelic/recessive disease trait genes.

### Meta-analysis suggests under-representation of the NAHR deletion alleles in currently discovered recessive disease trait allele pools

The striking prediction of the high contribution of NAHR deletions to relevant recessive disease trait load is seemingly contradictory to our current impression of the recessive allele landscapes. This implication led us to hypothesize that the NAHR deletion alleles are currently under-represented in disease characterization efforts. To test this latter hypothesis, we analyzed the distributions of a near-complete catalogue of currently discovered disease alleles in 181 patient families affected with one of the recessive traits whose carrier burden are predicted to be almost exclusively from NAHR-mediated large deletions (Fd > 70% from Table 2). The cohorts are assembled by meta-analysis of all literature reports for patients with the corresponding recessive disease trait disorder recorded in HGMD (version 2020.4), with the assumption that most patients, penetrant for the clinical disease entity, with these extremely rare recessive disease trait disorders characterized in research efforts are reported in the literature. *NPHP1*, the top-ranking gene from Table 2, is a well-characterized recessive trait “disease gene,” for which many research characterized patients may not result in published literature. Therefore, *NPHP1* is not included in these analyses because the literature-assembled meta-analysis cohort is unlikely to represent the natural disease allele composition (i.e., clinical practice) in the world.

It is expected that all patients with biallelic disease variants fall into three categories (1) HMZ: those affected with homozygous small variants possibly from a close- or distant- consanguineous relationship, (2) SNV+SNV: those affected with compound heterozygous small variants, and (3) NAHR deletion CNV+SNV (NAHRdelCNV+SNV): those affected with a large deletion in *trans* with a small variant allele. We anticipate that category #1-HMZ accounts for a substantial proportion, demonstrating the well-established robustness of autozygosity mapping as a method for allelic and new recessive trait gene discovery (as populational rare alleles can be escalated to much higher clan allele frequency) [14]. In outbred pedigrees and populations corresponding to categories #2-SNV+SNV and #3-NAHRdelCNV+SNV, our modeling from the NIRD hypothesis is that #3-NAHRdelCNV+SNV should account for a higher fraction. The opposing trend would suggest that our current disease gene/allele discovery efforts are not exploiting the large deletion allele to the fullest extent that a “human haploid genetics” approach might allow.

All the genes except for *RBM8A* show a poor representation for the #3-NAHRdelCNV+SNV configuration, based on the 104 families when excluding the ones affected with *RBM8A* variants from the entire cohort (Additional file 1: Table S6). Note that since most of the variants reported in these families are only documented once in affected human subjects, we cannot rule out the possibility that some of these variants are not causative to the clinical presentation, i.e., the variants of interest are not pathogenic determining alleles. More than two thirds (71/104) of these families carry homozygous disease alleles (57 unique alleles). Based on our modeling and the assumption of random mating, patients with homozygous variants are expected to account for a small fraction of the overall cohort, ranging from 0.53 to 5.4% per gene. However, the observed fractions of homozygotes for each gene are 1.9 to 189 (median 47) fold higher than expected. Furthermore, all of the 71 (or 57 unique) homozygous variants are rare, with 43 being ultra-rare (as defined by not observed in gnomAD v3.1). The collective patterns suggest that current efforts investigating these recessive traits tend to ascertain patients from populations with elevated autozygosity or from targeted population groups with their ethnic-specific disease founder alleles.

To avoid potential confounding factors from study designs and patient ascertainment methods, we removed patients with homozygous variants and focused on those with compound heterozygous variant alleles. Our modeling predicts that for these top-ranking genes analyzed, the number of patients with NAHRdelCNV+SNV should be 2.8 to 20 (median 10) fold higher than the number of patients with compound heterozygous small variants (Additional file 1: Table S6). The observed counts from many individual genes are too low to support a meaningful conclusion, but in aggregate, we have identified fewer patients with NAHRdelCNV+SNV [14] compared with patients with compound heterozygous small variants [16]. The recurrent deletions involved are 16p13.11 (*n*=4), 17p12-HNPP (*n*=3), DiGeorge 22q11.2 (*n*=2), 10q11.21q11.23 (*n*=2), 1q21.1-TAR (*n*=1), the Smith Magenis syndrome deletion (*n*=1), and proximal 16p11.2 (*n*=1). The poor representation of deletion-bearing patients shows a bias that under-represents category #3-NAHRdelCNV+SNV and deviates from the expectation driven by our analysis using empirical population allele frequencies and the NIRD score.

*RBM8A* is the only gene from our analysis that demonstrated a discovery pattern favoring #3-NAHRdelCNV+SNV, with the majority (95%, 73/77) of patients affected with the *RBM8A*- thrombocytopenia-absent radius (TAR; OMIM #274000) syndrome being compound heterozygous for the 1q21.1-TAR deletion and a small variant, whereas no patients were found to carry homozygous *RBM8A* pathogenic variants (Additional file 1: Table S6). This finding is consistent with expectations from our computational modeling based on the allelic spectra illustrating an overwhelming fraction of contribution of the 1q21.1 NAHR deletion at the disease locus (Tables 2 and S6). Moreover, the observed representation of NAHRdelCNV discovery at this locus, in contrast to other loci, is expected because of a unique characteristic of the *RBM8A*-1q21 locus. The disease presentation requires a combination of the rare 1q21 NAHR deletion null allele and a common (~1% minor allele frequency) hypomorphic small variant [48]. However, neither of the two allele types can be found in patients as homozygotes—the NAHR deletion homozygotes are lethal and the homozygous hypomorphic small variants are not disease-triggering. The unique molecular allele architecture and disease pathogenic mechanism of *RBM8A*, a condition that is clinically uniform and genetically homogeneous, shuts the door of discovery by sequencing of population with high autozygosity, but spontaneously presented the #3-NAHRdelCNV+SNV configurations for research discovery [49]. Similar expectations, empirical modeling and observations for *Tbx6-*derived scoliosis, i.e., *TBX6-*associated congenital scoliosis in mice, were found [26, 29].

### A human haploid genetics and genomics approach to recessive trait genes

We retrospectively analyzed two existing clinical cohorts to find data that test our computational prediction of NAHR deletions conferring a major disease burden to many recessive disease traits. The configurations of the two cohorts are not optimized for discovery, but seem to have provided preliminary evidence in support of our prediction from computational modeling. The first cohort was assembled focusing on the *COX10* gene, defects of which cause mitochondrial complex IV deficiency (OMIM# 220110) inherited as an AR trait.

*COX10* is located within the 17p12 recurrent deletion that is associated with hereditary neuropathy with liability to pressure palsies (HNPP, OMIM# 162500), a mild form of peripheral neuropathy, or a dominant susceptibility locus to neuropathy after traumatic injury, akin to an animal model observed as the Wallerian degeneration slow phenotype modeled in the *Wld* triplication mouse [50]. HNPP is due to decreased dosage of the *PMP22* gene via haploinsufficiency and is inherited as a liability to pressure palsies originally described in the Dutch population and pathologically presenting as tomaculous neuropathy [51, 52]; it is often only manifested clinically as multifocal neuropathy elicited after sustained trauma to a peripheral nerve that traverses close to the body surface and manifest as an entrapment neuropathy [53] or an operative carpal tunnel syndrome co-segregating through multiple generations [27]. *PMP22* maps within the 1.5 Mb HNPP deletion CNV and *COX10* is the only gene in the deletion interval with a known AR disease trait association other than *PMP22*; the latter *PMP22* is associated with both an AD and AR neuropathy traits [54, 55]. Based on our calculation, ~77% of all patients affected with biallelic *COX10* pathogenic alleles in an outbred population carry one HNPP deletion (Table 2).

We retrospectively investigated results from 596 patients suspected with a mitochondrial disorder who were clinically tested for *COX10* coding region sequencing and deletion/duplication CNV analyses. The strength of the patient ascertainment strategy from this cohort is that patients were referred based on clinical suspicion, and therefore the distribution of pathogenic alleles from this cohort is likely free of a “molecular diagnosis bias.” A weakness of this cohort configuration is that the selected disease phenotype is of high genetic heterogeneity, which inherently predicts that only a small number of patients will indeed be affected with a *COX10*-related condition. Nevertheless, we found two patients received a possible molecular diagnostic finding in *COX10*, both carrying the HNPP deletion as one allele.

In subject #1, a hemizygous variant resulting in an in-frame small duplication of two amino acids, c.1277_1282dup (p.M426_L427dup) in exon 7 of *COX10*, was identified in *trans* to the HNPP deletion (Additional file 3: Figure S2). Subject #2, whose referral indication is COX deficiency, has a rare VUS c.858G>T (p.W286C) in *COX10* in *trans* with the HNPP deletion. In the remaining patients without a definitive molecular diagnosis, two patients were found to have the heterozygous HNPP deletions, but a second hit in *COX10* was not found, although we cannot rule out the possibility of additional findings in intronic or regulatory regions. These findings, though under-powered, are consistent with our prediction that most patients with cytochrome c oxidase deficiency carry one HNPP deletion allele, either de novo or inherited. Considering the high frequency of the HNPP susceptibility allele [20] with absence of selection and late-onset adult disease [56], it is possible that more novel *COX10* disease alleles can be revealed by sequencing individuals with the HNPP deletion and a mitochondrial spectrum of clinical phenotypes, thereby improving our understanding of the biological function of the *COX10* gene.

The second cohort we assembled is based on the criteria that a patient carries one of the NAHR deletions and that genotype information of the non-deleted allele is available for analyses. Thus, we identified such individuals from a cohort of 11,091 subjects who were referred for clinical exome sequencing (cES) at a diagnostic laboratory due to a differential clinical diagnosis including various suspected genetic disorders. We performed an initial screen for patients carrying one of the genomic deletions from Table 1, which resulted in 161 subjects carrying one recurrent deletion and 3 subjects carrying two. The two most frequently observed types of deletions, the 15q11.2 BP1−BP2 deletion (*n*=41) and the *NPHP1*-2q13 deletion (*n*=23), are excluded from downstream analysis. This exclusion is because none of the coding genes from the 15q11.2 BP1−BP2 deletion have been implicated to be associated with a Mendelian disease trait [57], and the critical gene at 2q13, *NPHP1*, has been already extensively studied [58]. We also excluded six subjects harboring the X-linked, hemizygous deletion in the Xp22.31 *STS* locus. After excluding these three groups of deletion CNVs, cES data from personal genomes of 95 subjects, collectively harboring 96 incidences or 26 types of recurrent genomic deletions, were available for us to build the second cohort (Additional file 1: Table S7).

This second cohort is not optimized for discovery because it is a collection of various different deletions without any enrichment for a targeted phenotype. Additionally, despite a subset of these patients carry one of the disease-associated large deletions that are known before cES, they are still referred for cES analyses; such a property predicts that the disease pathogenesis mechanism found in this cohort tend to be more complex than a typical Mendelian disease cohort. Such individuals may more likely to be represented by a “blended phenotype” [59].

Again, in accordance with our expectations, more than a quarter (26/95) of these subjects were found to have probable small variant molecular diagnostic findings independent from the deletion. From the remaining 69 subjects with an apparent undiagnostic cES result, we identified 4 subjects with rare variants in coding regions exposed by the deletion as potential molecular diagnoses (Table 3). The first patient is subject #1 described above with HNPP deletion and a *COX10* small variant allele.

**Table 3:** Clinically significant sequence variants uncovered by the deletions. Subjects #1 and #2 were identified in a *COX10*-phenotype-driven cohort analysis. Subjects #1, #3, and #4 were identified in the molecular-deletion-driven clinical exome data reanalysis

ID	Deletion/ allele frequency	Gene (RefSeq transcript)	Genic variant	Genomic coordinate (GRCh38)	MAF in gnomAD v3.1	Classification	Category
**1**	17p12 HNPP/ 3.148 × 10^−4^	*COX10* (NM_001303.3)	c.1277_1282dup (p.M426_L427dup)	chr17:14207158_ 14207163dup	0	VUS	NDAC
**2**	17p12 HNPP/ 3.148 × 10^−4^	*COX10* (NM_001303.3)	c.858G>T (p.W286C)	chr17:14192151G>T	6.567 × 10^−6^	VUS	NDAC
**3**	10q11.21q11.23 deletion/ 1.353 × 10^−4^	*ERCC6* (NM_000124.3)	c.1490T>C (p.F497S)	chr10:49505920A>G	0	VUS	NDAC
**4**	15q13.3 BP4-BP5 deletion/ 9.97 × 10^−5^	*OTUD7A* (NM_130901.2)	c.2023_2066del (p.D675Hfs*188)	chr15:31484009_ 31484052del	6.58 × 10^−5 a^	VUS	NDGMC
**5**	16p11.2 proximal / 5.076 × 10^−4^	*PRRT2* (NM_145239.2)	c.649dup (p.R217fs*8)	chr16: 29813703dup	1.472 × 10^−4 b^	Pathogenic	NDGMC

^a^This variant is marked with “low complexity region” label in gnomAD, suggesting ambiguous variant call quality. It has a variant count of 2 in gnomAD v3.1. However, manual review of the alignment data from gnomAD suggests only 1 is of higher quality. The allele frequency is adjusted in half accordingly

^b^This variant is marked with “low complexity region” label in gnomAD, suggesting ambiguous variant call quality. It is located in a homopolymer region that is susceptible to false positive variant calling. The variant allele frequency quoted here may be overestimated

Abbreviations: *MAF*, minor allele frequency; *VUS*, variant of unknown clinical significance; *NDAC*, new disease allele characterization; *NDGMC*, new disease gene/mechanism characterization

The second patient, subject #3, has clinical features including ataxia, developmental delay, microcephaly, and short stature. A recurrent 10q11.21q11.23 deletion [60] was identified in *trans* to a novel missense variant allele c.1490T>C (p.F497S) in the *ERCC6* gene. Biallelic variants in *ERCC6* are associated with cerebro-oculo-facio-skeletal syndrome 1 (COFS1, MIM# 214150) or Cockayne syndrome type B (CSB, MIM# 133540). The high allele frequency of the 10q11.21q11.23 deletion (1.412×10^−4^) increases the probability for a second allele with ultra-low frequency, like the c.1490T>C (p.F497S) *ERCC6* variant, to be correlated with a set of human clinical phenotypes.

Subject #4 presented with severe neurodevelopmental diseases and dysmorphic features. We identified a hemizygous *OTUD7A* frameshift variant allele c.2023_2066del (p.D675Hfs*188) in *trans* with the recurrent 15q13.3 BP4-BP5 deletion, providing evidence for *OTUD7A* as a new disease gene. The recurrent deletion mediated by BP4 and BP5 at the 15q13.3 locus is associated with highly variable NDD (neurodevelopmental disorder) phenotypes, ranging from asymptomatic to mild to moderate intellectual disability, epilepsy, behavioral issues distinct from neurotypical behaviors (e.g., autism spectrum disorders, attention deficit hyperactivity disorders), and variable dysmorphic features [61, 62]. While heterozygous deletion causes highly variable phenotypes, reported homozygous 15q13.3 BP4-BP5 deletion consistently manifest disease phenotypes including significant NDD, epilepsy, hypotonia, visual impairments, and other less common phenotypes including autism spectrum disorder, short stature, failure to thrive, microcephaly, and variable dysmorphic features (Additional file 1: Table S8) [63–67]. The critical gene responsible for this “ciliopathy like clinical presentation” of the 15q13.3 BP4-BP5 deletion has been debated, but evidence suggests that *OTUD7A*, encoding a member of a family of deubiquitinating enzymes, may be a plausible candidate [68, 69].

Studies using syntenic heterozygous deletion mouse models suggest a critical role of Otud7a in neuronal development and brain function [68, 69]. Otud7a-null mouse models manifest many cardinal features of the 15q13.3 deletion syndrome [68]. The c.2023_2066del (p.D675Hfs*188) variant identified in subject #4 maps to the last exon of the *OTUD7A* gene, and is thus predicted to not result in nonsense-mediated decay (NMD) [70]. However, the variant is predicted to result in substitution of the C-terminal amino acids after aspartic acid with 187 novel amino acids and a premature termination of the protein translation (PTC). This change may remove the C-terminal Zinc finger A20-type domain and abolish the normal function of the protein. Our finding in Subject #4, together with recent case reports of patients with a homozygous missense *OTUD7A* variant alleles [71], or compound heterozygous 15q13.3 deletion in *trans* with a frameshift *OTUD7A* variant [72], supports our contention and corroborates that *OTUD7A* may be the critical “driver gene” in the 15q13.3 deletion syndrome. *OTUD7A* may be sensitive to gene dosage effect and contribute to disease etiology at least in part through a biallelic AR disease trait mechanism.

Interestingly, we observe that the population small variant allele pool for *OTUD7A* is depleted for LoF alleles based on gnomAD. Without the 15q13.3 deletion contributing to a major carrier burden, the paucity of small variant disease alleles for *OTUD7A* would make disease association establishment using patient data much more challenging. From an alternative perspective, *OTUD7A*’s current apparent “high” gene intolerance to haploinsufficiency (pLI=0.95) may have incidentally portrayed it as a “dominant” Mendelian disease gene, whereas the calculated high NIRD score (5.3) of the gene strongly indicates that the intolerance to haploinsufficiency should be much lower, i.e., low likelihood of being an AD trait gene.

In Subject #5 with severe NDD, we identified a c.649dup (p.R217fs*8) pathogenic variant in the *PRRT2* gene in *trans* with the recurrent 16p11.2 BP4-BP5 deletion, providing compelling evidence for a novel disease AR trait inheritance mechanism for *PRRT2*. The 16p11.2 BP4-BP5 recurrent deletion is known to be associated with mild dysmorphisms, macrocephaly, and neuropsychiatric phenotypes including DD/ID and autism spectrum disorder (ASD) with incomplete penetrance, a NDD [73, 74].

The *PRRT2* gene is highly expressed in mouse brain and spinal cord during early embryonic development [75]. Heterozygous LoF variants in *PRRT2* cause movement and seizure disorders including familial infantile convulsions with paroxysmal choreoathetosis (OMIM# 602066), episodic kinesigenic dyskinesia 1 (EKD1, OMIM# 128200), or benign familial infantile seizures 2 (BFIS2, OMIM# 605751), with incomplete penetrance documented [76]. The c.649dup (p.R217fs*8) allele is the most frequent pathogenic variant, occurring at a mutational hotspot with homopolymer of 9 cytosine bases adjacent to 4 guanine bases that are susceptible to DNA replication errors [77]. Currently, autosomal dominant (AD) is considered as the only disease inheritance mode for *PRRT2* traits in OMIM, although preliminary evidence from case reports suggest that *PRRT2* can cause a more severe NDD through a biallelic pathogenic mechanism and an AR inheritance model [78]. Our findings in Subject #5 provide further support for the contention of a new rare disease trait type, AR versus AD, and inheritance mechanism due to *PRRT2* biallelic variation. Moreover, these observations may also highlight a potential compound inheritance gene dosage (CIGD) model that explains penetrance of certain neurological phenotypes observed in patients with the 16p11.2 deletion; a similar biallelic compound inheritance gene dosage model underlies the penetrance of ~10–12% of all congenital scoliosis worldwide [79].

### NAHR deletions contribute to recessive disease burden in population-specific patterns

As suggested earlier, the contribution of a given allele to rare recessive disease trait burden is influenced by the composition of other pathogenic alleles from the same gene. Although the genetics and genomics fields are beginning to appreciate inter-individual variabilities in NAHR rates associated with alternative genomic structural haplotypes [26, 58, 80] as well as polymorphisms from *trans* acting factors controlling homologous recombination, such as *PRDM9* [20], we currently still assume that NAHR mutation rates at a given locus are relatively constant across different populations and genomic ethnic backgrounds. This potentially leaves the remaining alleles, the small variants, as the major driver for any variability in allelic architecture from different population groups.

**Fig. 3:**
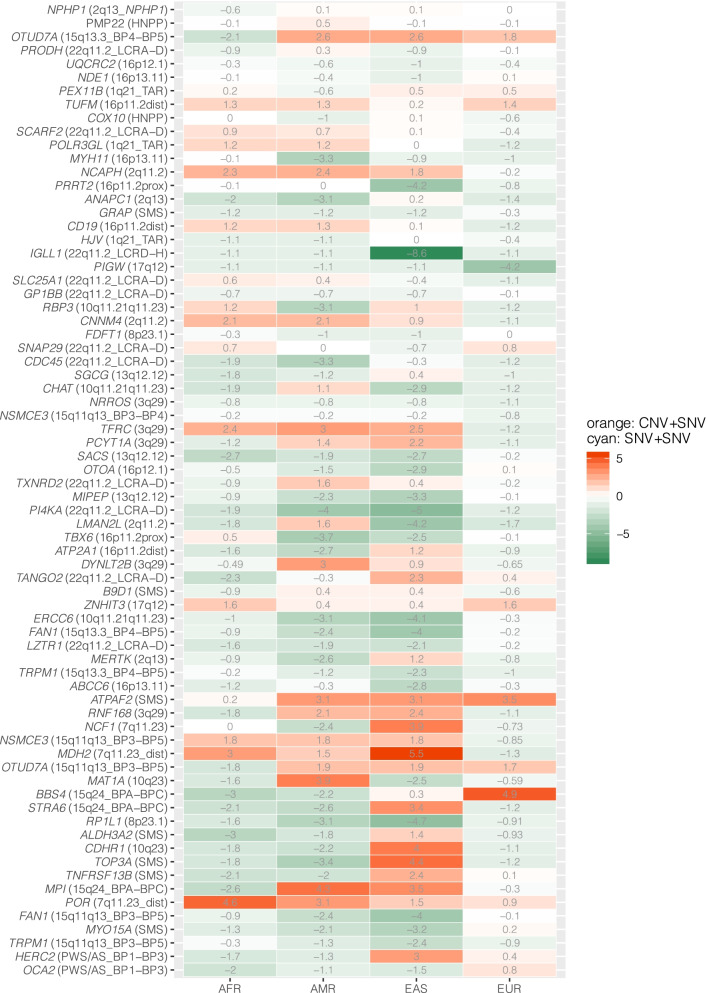
ΔNIRD scores for a population group relative to the general population. ΔNIRD is calculated by subtracting the general population NIRD from the specific population group NIRD. A positive ΔNIRD score suggests an even higher recessive disease contribution of the NAHR deletion from that population group. A negative ΔNIRD score indicates a relatively higher probability for an affected patient to carry biallelic small variants in that population group. Genes that have zero ΔNIRD scores for all four population groups are not depicted in this heatmap

To investigate the degree of inter-population variability for small variant recessive alleles, we used ethnic information from gnomAD and conducted the modeling described earlier for four population groups, African (AFR), Latino (AMR), East Asian (EAS), and European (EUR) (Additional file 1: Table S5). Population-specific NIRD scores are compared with the general population to generate ΔNIRD (Fig. 3), which can be used to inform the relative odds ratio for NAHR deletions in rare AR disease traits in the specific populations. These analyses provide preliminary computational confirmation for the suspected population variability in NIRD, which implicates that the precision of NIRD can be improved by “tuning the disease model” with population-specific allelic architecture. In light of these surprising findings, we tentatively propose, i.e., we hypothesize, that a prioritization strategy based on prior knowledge of population allele frequency spectra can be applied to enhance discovery in research study design of genomic sequencing among individuals with large recurrent deletions. We cautiously note that some of the population groups analyzed here may not have a sufficient sample size to allow a complete representation of disease alleles of relatively lower frequency, which may result in overestimation of ΔNIRD when the score is positive. Additional population-specific allele frequency data are warranted to improve the accuracy of these analyses.

## Discussion

We cataloged the population frequency spectra of pathogenic alleles across all known AR disease traits. Our analyses highlighted the high frequency of SV mutagenesis and NAHR deletion alleles in genomic instability regions susceptible to this type of structural variant mutagenesis-mediated rearrangement [81]. We subsequently computationally interrogated the impact of allele frequency distributions on recessive disease trait burden using a modified Punnett square. We used this computational and genetic approach to consider the impact of the NAHRdelCNV allele on recessive disease traits relative to other recessive trait biallelic variants. Our findings indicate the potentially dramatic disease impact of these NAHRdelCNV—a group of new mutation alleles resulting from recurrent rearrangement; our findings have profound implications for disease biology and molecular diagnosis worldwide.

Considering current clinical genomics practice and research investigations, we postulate that the role of recurrent genomic deletions and new mutation contributing to recessive diseases is under-appreciated, therefore potentially impeding discovery of new disease genes and alleles. Traditionally, researchers and clinicians tend to consider large causative genomic deletions as dominant disease trait halpoinsufficient alleles, with the driver dosage-sensitive gene(s) mapping within the deleted interval [22]. This haploinsufficiency assumption likely arose because these alleles were almost all identified as heterozygotes by screening in symptomatic cohorts and frequently as de novo CNV mutations. Our analysis of population frequency implicates that the heterozygous state is often not disease-causing, and therefore that these variants act as contributors to recessive disease when they occur in *trans* to an AR disease trait allele.

Although many recurrent deletions contribute considerable carrier allele burden to individual recessive Mendelian disease traits, these deletions are often large enough to include other genes whose homozygous depletions are incompatible with live birth. Two exceptions are the 2q13-*NPHP1* deletion and the *SMN1* deletion (the *SMN1* deletion CNV, observed to be found as a carrier state in outbred populations, is predicted by our analyses to be the most frequent NAHR-mediated deletion, NAHRdelCNV allele as shown in Additional file 1: Table S2). The fact that other large recurrent deletions are almost never observed in patients as homozygous losses may have led investigators to overlook their equally important role in contributing to recessive disease traits as compound heterozygotes [82].

Clinically, there has not been a consensus on whether exome/genome sequencing should be pursued after a recurrent genomic deletion has been identified in a patient [83, 84]. Some may argue that, under the assumption that most Mendelian diseases are caused by one “unifying diagnosis,” the identification of a large genomic deletion can be evidence to “demote” additional candidate molecular diagnoses in the same patient. Our data argue the opposite: that in patients with a recurrent “contiguous gene deletion syndrome,” the possibility of revealing an “additional” recessive disease trait molecular diagnosis cannot be ignored.

Of note, four of the five hemizygous small variants exposed by the deletion in Table 3 are located in repeat or difficult-to-sequence regions. The *COX10* and the *PRRT2* frameshift variants were incorrectly “called” as heterozygous changes by the original exome variant calling pipeline due to challenges of calling indels and potentially other “mappability” issues inherent to such regions of the human genome. If heightened diligence for variants falling in these difficult to assay variation regions were not taken to examine specifically look into the deleted hemizygous interval, these molecular diagnostic variants could have easily been missed during analyses. We suggest this contention might be one of the reasons contributing to the proposed under-detection of recurrent deletion (i.e., NAHRdelCNV) + small variant cases for recessive trait disorders.

Of note, the NIRD score devised in this study not only directly depicts the NAHRdelCNV contribution to the gene’s rare recessive trait disease load, but also highlights features not captured by the haploinsufficiency tolerance score pLI. Of the 48 genes with a high NIRD (>3.5) score, 6 have high pLI scores (>0.9) predicting intolerance to haploinsufficiency. These pLI estimations are not accurate because the calculations failed to factor in the population prevalence of NAHR deletion alleles. Gene-level constraint scores should be adjusted particularly for the high NIRD genes.

**Fig. 4:**
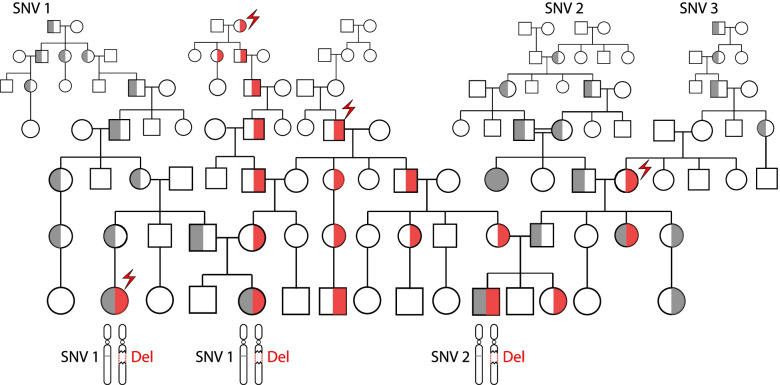
A “segmental haploid genomics” approach for characterization of new disease genes or alleles for autosomal recessive conditions. The illustration above depicts dynamics of disease alleles in an autosomal recessive condition whose collective carrier burden is contributed by a recurrent genomic deletion (red area) and a few single-nucleotide variants (SNVs, gray area). Individuals affected with biallelic pathogenic changes frequently carry the deletion as one of the two alleles, because the deletion arises recurrently in multiple lineages (indicated by the red lightning bolt arrow). SNV disease alleles for recessive genes tend to be passed on from ancestral generations and may drift away without being noticed if they do not converge with another disease allele (SNV3). However, they may emerge to medical attention frequently in families with high degree of autozygosity, as illustrated in generations 3 and 4 for SNV2

Taken together, we suggest that the future of new recessive disease trait genes and allele discovery will greatly benefit from two approaches: (1) genomic sequencing of individuals with recurrent deletions (NAHRdelCNV) and (2) sequencing in population of elevated autozygosity (Fig. 4). The autozygosity mapping approach has been the classic approach for new disease gene discovery in medical genetics [13, 14, 85]. This approach enables an adept strategy to target patient populations (by assessing their degree of autozygosity from social and family histories) in order to assemble the appropriate cohorts for research investigations. However, disease trait alleles revealed by this approach are usually limited to a specific population [13, 14, 85].

The NAHR deletion sequencing approach, on the other hand, capitalizes on the known high mutation rates of NAHRdelCNV and has the potential to assign clinical significance to alleles independent of ethnic backgrounds, this latter contention at least partly owing to the recurrent and high mutation rate of the NAHR SV mutagenesis mechanism. Nevertheless, this approach requires prior knowledge and screening of individuals for the recurrent deletion CNVs. It may have been challenging to collect a cohort of patients with large recurrent deletions two decades ago, but the “clinical awareness” of genomic disorders [2, 52] and advancements in clinical testing and populational screening have made such a genomic experimental effort feasible, either from large diagnostic centers or from clinical registries [25, 30, 57, 73, 74, 86, 87]. Even when available subject numbers are limited for recurrent genomic deletion CNVs with extremely low penetrance, it is possible to tune the disease gene/allele characterization strategy by targeting specific phenotypes, as demonstrated at the Smith Magenis Syndrome - *MYO15* locus two decades ago [88].

Whilst researchers are starting to sequence cohorts of individuals with large “Mb-sized” deletions [21, 89, 90] and performing SNV and CNV analysis in one combined WGS assay, it is imperative for clinical and diagnostic genomicists to foster guidelines that facilitate routine genomic sequencing (ES or WGS) on patients who are found to have recurrent genomic deletions, i.e., NAHRdelCNV, that will benefit both the patients and the research human subjects worldwide. Given the great potential in the near future for disease gene discoveries within intervals of genomic disorder deletion CNVs, these patients will benefit from routine or perhaps even more prioritized reanalysis of sequencing data [91].

Performing DNA sequencing on personal genomes with higher population prevalence of recurrent deletions (those from Table 1) carries additional long-term promise for clinical characterization of common variant alleles, extending the current scope of focus on mono- or biallelic inheritance into the more complex spectrum of disease inheritance—including the compound inheritance gene dosage model, CIGD [26]. High prevalence recurrent genomic deletions are often associated with a high degree of incomplete penetrance of disease phenotype, ranging from 10 to 90% [22]. The disease causal mechanism described in this study, for example, the 16p11.2 deletion + the *PRRT2* small variant leading to NDD, may explain a small portion of the previously attributed missing heritability for the disease “penetrance” at 16p11.2. However, the totality of the missing heritability is likely not explained by the recessive trait model alone, because the observed disease penetrance is likely to exceed the aggregation of the recessive disease allele prevalence (individually rare) on the non-deleted chromosome. It is plausible that alternative disease models exist, in which the critical gene triggers disease presentation when one rare LoF allele is combined with one or a set of milder hypomorphic alleles with common population frequency. This compound inheritance model has been demonstrated at the *RBM8A*-1q21.1 locus in association with the TAR syndrome [48], the *TBX6*-16p11.2 locus in association with congenital scoliosis [26, 29], the *F12*-5q35 Sotos deletion locus in association with blood clotting [92], and the *TBX4-FGF10* lung disease [93].

Our data and analyses in this study were focused on coding sequence changes. Moreover, the limited size of patient cohort ascertained for each recurrent deletion CNV may decrease the power of identifying high-frequency hypomorphic alleles. These will be dramatically empowered by genome sequencing in a larger patient cohort. Nevertheless, the unifying theme for both the strictly recessive model, and the more complex compound inheritance gene dosage model, is that large recurrent, NAHR-derived, genomic deletion CNVs, i.e., NAHRdelCNV alleles often associated with a genomic disorder when heterozygous [52], may provide a unique perspective in characterization of new disease trait loci and alleles, the biology of disease, and the emerging field of human haploid genetics.

## Conclusions

We have demonstrated through computational modeling that NAHR-mediated recurrent genomic deletions contribute to a major fraction of burdens for recessive disease traits, for 74% of loci within these segmental deletions or at least 2% of loci genome-wide. Our meta-analysis over literature data regarding recurrent deletions suggests that the sequencing effort of these personal genomes is currently under-appreciated. We propose that systematic sequencing of individuals carrying NAHR-mediated recurrent genomic deletions is a promising genomic strategy for discovery and characterization of autosomal recessive disease trait genes and alleles.

## Supplementary Information

**Additional file 1: Table S1.** All clinically reported recurrent deletions and their prevalence estimates. **Table S2.** All 717 recurrent genomic deletions predicted based on the repeat structure in the human reference genome GRCh38. **Table S3.** Carrier disease allele frequencies by allele. **Table S4.** Carrier allele frequency burden by gene. The list is ranked by genes from the highest burden to the lowest burden. The frequency burden in this list only includes the actual observed variants; the 10% extra hypothetical uncharacterized alleles as described in the Methods section are not included. **Table S5.** Gene-level NAHR contribution to carrier allele and recessive disease burden as well as NAHR Deletion Impact to Recessive Traits (DIRT). This table is comprised of five panels, representing results generated using data from the general population and four specific ethnic groups, including African (AFR), Latino (AMR), East Asian (EAS), and European (EUR). **Table S6.** Meta-analyses for literature reported patients affected with the 13 recessive disorders contributed by significant NAHR-mediated deletion burden. Compound heterozygous variants are split into two rows with each row representing one variant. **Table S7.** Molecular findings of recurrent deletions identified from clinical exome sequencing. **Table S8.** Literature review for 15q13.3 recurrent deletions.

**Additional file 2: Supplementary Methods**: Calculation of NAHR-deletion contribution to disease burden for a specific recessive disorder: modified model specific for *NPHP1*-2q13, 15q13.3 BP4-BP5, *RBM8A*-1q21.1 and *TBX6*-16p11.2.

**Additional file 3: Figure S1.** Genome-wide map for all predicted NAHR recurrent genomic deletions. Each predicted deletion event is marked as a green horizontal bar below the chromosome ideograms. The vertical bars above the chromosome ideograms illustrates the density for segmental duplications in a 1000-bp moving window. **Figure S2.** Compound heterozygous HNPP deletion and *COX10* variant leading to recessive COX10 deficiency in Subjects #2 and #3. **A.** The *COX10* gene spans the repeat sequence that mediate the recurrent HNPP deletion at chromosome 17p12. The *COX10* variant in the Subject #2 is located at the 3’ end of the *COX10* gene on exon 7, which is inside the HNPP deletion interval. The *COX10* variant in the Subject #3 is located at the *COX10* gene exon 7, which is embedded in a CMT1A-REP. Red segments, exons of the *COX10* gene; yellow arrows, CMT1A-REPs; thunderbolts, *COX10* variants observed in Subject #2 and #3, respectively. **B.** Diagram illustrating the scheme of the relationship between the SNV/INDEL and recurrent deletion identified in Subject #2 and #3.

## Data Availability

Detailed code for generation of the NAHR deletion map, preparation of disease trait alleles, and calculation of disease burden is available at GitHub [94] (https://github.com/liu-lab/cnvNAHR/). All data generated are available in the manuscript and its supporting files.
