# Supplementary material for: Sequencing individual genomes with recurrent genomic disorder deletions: an approach to characterize genes for autosomal recessive rare disease traits
Source: Genome Med. 2022 Sep 30;14:113. doi: 10.1186/s13073-022-01113-y (PMC9526336; doi:10.1186/s13073-022-01113-y)
Supplement: Supplementary file 2 — Additional file 2: Supplementary Methods: Calculation of NAHR-deletion contribution to disease burden for a specific recessive disorder: modified model specific for NPHP1-2q13, 15q13.3 BP4-BP5, RBM8A-1q21.1 and TBX6-16p11.2. [file 13073_2022_1113_MOESM2_ESM.docx]

Supplementary Methods

**Calculation of NAHR-deletion contribution to disease burden for a specific recessive disorder: modified model specific for *NPHP1*-2q13*,* 15q13.3 BP4-BP5, *RBM8A*-1q21.1 and *TBX6*-16p11.2**

All the following calculations are based on the concept of random mating by sampling from a pool of alleles. Suppose that at an autosomal recessive locus, we have *n* alleles, A_1_, A_2_, … , A_h_, A_k_, A_l_, …, A_n_, with allele frequencies *p_1_*, *p_2_*, …, *p_h_*, *p_k_*, *p_l_*, …, *p_n_*. Without loss of generality, we nominate the k^th^ allele as the NAHR-deletion allele -our allele of interest - and let the others index the small variant alleles. *l* denotes NAHR-deletion homozygous lethal alleles. For most large recurrent deletions, where homozygous loss of the deletion is incompatible with live birth, A_l_ denotes the same allele as A_k_; in conditions when homozygous deletions are compatible with live birth, allele A_l_ either does not exist (2q13-*NPHP1* deletion) or refers to an alternative NAHR-deletion allele which combined with the NAHR-deletion allele of interest is lethal (for the 15q13.3 BP4-BP5 deletion, its A_l_ is the 15q11q13 BP3-BP5 deletion). *h* denotes hypomorphic alleles. In our modeling, hypomorphic alleles only exist for 1q21.1-*RBM8A* and 16p11.2-*TBX6*. Hypomorphic alleles are distinctive from other LoF alleles in that biallelic hypomorphic alleles are not disease causing.

We denote the probability of an individual carrying the NAHR-deletion allele is

The contribution of the NAHR-deletion to the allele load (**Fa**) is the fraction of the NAHR-deletion allele frequency over the sum of all allele frequencies across all functional alleles - except for the hypomophric alleles that do not in themselves cause disease.

The Punnett square below models the expected frequencies of an individual to be affected with the recessive trait given the population frequencies of each pair of alleles under a random mating model.

|  | A_1_  *p_1_* | A_2_  *p_2_* | … | A_k_  *p_k_* | *…* | A_n_  *p_n_* |
| --- | --- | --- | --- | --- | --- | --- |
| A_1_ *p_1_* | ***p_1_*^2^ A_1_A_1_** | ***p_1_p_2_* A_1_A_2_** | **…** | ***p_1_p_k_* A_1_A_k_** | **…** | ***p_1_p_n_* A_1_A_n_** |
| A_2_ *p_2_* | ***p_1_p_2_* A_1_A_2_** | ***p_2_*^2^ A_2_A_2_** | **…** | ***p_2_p_k_* A_2_A_k_** | **…** | ***p_2_p_n_* A_2_A_n_** |
| *…* | ***…*** | ***…*** | ***…*** | ***…*** | ***…*** | ***…*** |
| A_k_ *p_k_* | ***p_1_p_k_* A_1_A_k_** | ***p_2_p_k_* A_2_A_k_** | ***…*** | ***p_k_*^2^ A_k_A_k_** | **…** | ***p_k_p_n_* A_k_A_n_** |
| *…* | ***…*** | ***…*** | **…** | ***…*** | **…** | ***…*** |
| A_n_ *p_n_* | ***p_1_p_n_* A_1_A_n_** | ***p_2_p_n_* A_2_A_n_** | **…** | ***p_k_p_n_* A_k_A_n_** | ***…*** | ***p_n_*^2^ A_n_A_n_** |

The probability for an individual to be affected with the recessive disorder is the sum of pairwise products of all carrier alleles with contribution from biallelic hypomorphic alleles (*h*) and homozygous NAHR-deletion lethal allele (*l*) subtracted

The probability for an individual to be both affected with the recessive disorder and carrying the NAHR-deletion is

The contribution of individuals with the NAHR-deletion to the recessive disease load (**Fd**) is

The odds for an individual with the recessive disease to carry the NAHR deletion is

Note that the sum of Fd across alleles *1*, *2*, *3*, …, *n* equals the sum of the Punnett square matrix plus the lower triangular and the upper triangular. This is equivalent to summing up homozygous allele products once plus compound heterozygous allele products twice. This characteristic arises because the events of recessive disease involving the k^th^ and the j^th^ allele are not disjoint and they overlap for the compound heterozygote entries in the Punnett square.

To calculate the NAHR-deletion Impact to Recessive Disease score, the odds of the NAHR allele is compared to that of the “median” allele from the same gene.

The “median” allele is defined as the midpoint of remaining alleles in the same gene that comprise a cumulative sum of the top 90% of the overall sum of Fd. The alleles consisting the lower 10% of overall Fd sum are disregarded because (1) we find many genes have a long tail of ultra-rare alleles without a frequency estimate from gnomAD, and (2) we supplemented 10% of hypothetical alleles to each gene in our analysis.
