# Supplementary material for: Sequencing individual genomes with recurrent genomic disorder deletions: an approach to characterize genes for autosomal recessive rare disease traits
Source: Genome Med. 2022 Sep 30;14:113. doi: 10.1186/s13073-022-01113-y (PMC9526336; doi:10.1186/s13073-022-01113-y)
Supplement: Supplementary file 3 — Additional file 3: Figure S1. Genome-wide map for all predicted NAHR recurrent genomic deletions. Each predicted deletion event is marked as a green horizontal bar below the chromosome ideograms. The vertical bars above the chromosome ideograms illustrates the density for segmental duplications in a 1000-bp moving window. Figure S2. Compound heterozygous HNPP deletion and COX10 variant leading to recessive COX10 deficiency in Subjects #2 and #3. A. The COX10 gene spans the repeat sequence that mediate the recurrent HNPP deletion at chromosome 17p12. The COX10 variant in the Subject #2 is located at the 3’ end of the COX10 gene on exon 7, which is inside the HNPP deletion interval. The COX10 variant in the Subject #3 is located at the COX10 gene exon 7, which is embedded in a CMT1A-REP. Red segments, exons of the COX10 gene; yellow arrows, CMT1A-REPs; thunderbolts, COX10 variants observed in Subject #2 and #3, respectively. B. Diagram illustrating the scheme of the relationship between the SNV/INDEL and recurrent deletion identified in Subject #2 and #3. [file 13073_2022_1113_MOESM3_ESM.docx]

**Figure S1. Genome-wide map for all predicted NAHR recurrent genomic deletions.** Each predicted deletion event is marked as a green horizontal bar below the chromosome ideograms. The vertical bars above the chromosome ideograms illustrates the density for segmental duplications in a 1000-bp moving window.

**Figure S2.** Compound heterozygous HNPP deletion and *COX10* variant leading to recessive COX10 deficiency in Subjects #2 and #3. **A.** The *COX10* gene spans the repeat sequence that mediate the recurrent HNPP deletion at chromosome 17p12. The *COX10* variant in the Subject #2 is located at the 3’ end of the *COX10* gene on exon 7, which is inside the HNPP deletion interval. The *COX10* variant in the Subject #3 is located at the *COX10* gene exon 7, which is embedded in a CMT1A-REP. Red segments, exons of the *COX10* gene; yellow arrows, CMT1A-REPs; thunderbolts, *COX10* variants observed in Subject #2 and #3, respectively. **B.** Diagram illustrating the scheme of the relationship between the SNV/indel and recurrent deletion identified in Subject #2 and #3.
